# Age-specific differences in the time-frequency representation of surface electromyographic data recorded during a submaximal cyclic back extension exercise: a promising biomarker to detect early signs of sarcopenia

**DOI:** 10.1186/s12984-020-0645-2

**Published:** 2020-01-28

**Authors:** R. Habenicht, G. Ebenbichler, P. Bonato, J. Kollmitzer, S. Ziegelbecker, L. Unterlerchner, P. Mair, T. Kienbacher

**Affiliations:** 1 Karl-Landsteiner-Institute of Outpatient Rehabilitation Research, Vienna, Austria; 2 Department of Physical Medicine, Rehabilitation and Occupational Medicine, Medical University of Vienna, General Hospital of Vienna, Währinger Gürtel 18-20, 1090 Vienna, Austria; 3 Department of Physical Medicine and Rehabilitation, Harvard Medical School, Spaulding Rehabilitation Hospital, Boston, MA USA; 4 Technical School of Engineering, Vienna, Austria; 5 Department of Psychology, Harvard University, Cambridge, MA USA

**Keywords:** Aging, Surface electromyography, Muscle function, Muscle fatigue, Concentric exercise, Eccentric exercise

## Abstract

**Purpose:**

Motivated by the goal of developing new methods to detect early signs of sarcopenia, we investigated if surface electromyographic (SEMG) data recorded during the performance of cyclic, submaximal back extensions are marked by age-specific differences in their time and frequency characteristics. Furthermore, day-to-day retest reliability of the EMG measures was examined.

**Methods:**

A total of 86 healthy volunteers used a back dynamometer to perform a series of three maximal voluntary contractions (MVC) consisting of isometric back extensions, followed by an isometric back extension at 80% MVC, and finally 25 slow cyclic back extensions at 50% MVC. SEMG data was recorded bilaterally at L1, L2, and L5 from the iliocostalis lumborum, longissimus, and multifidus muscles, respectively. Tests were repeated two days and six weeks later. A linear mixed-effects model with fixed effects “age, sex, test number” and the random effect “person” was performed to investigate age-specific differences in both the initial value and the time-course (as defined by the slope of the regression line) of the root mean square (RMS-SEMG) values and instantaneous median frequency (IMDF-SEMG) values calculated separately for the shortening and lengthening phases of the exercise cycles. Generalizability Theory was used to examine reliability of the EMG measures.

**Results:**

Back extensor strength was comparable in younger and older adults. The initial value of RMS-SEMG and IMDF-SEMG as well as the RMS-SEMG time-course did not significantly differ between the two age groups. Conversely, the IMDF-SEMG time-course showed more rapid changes in younger than in older individuals. Absolute and relative reliability of the SEMG time-frequency representations were comparable in older and younger individuals with good to excellent relative reliability but variable absolute reliability levels.

**Conclusions:**

The IMDF-SEMG time-course derived from submaximal, cyclic back extension exercises performed at moderate effort showed significant differences in younger vs. older adults even though back extension strength was found to be comparable in the two age groups. We conclude that the SEMG method proposed in this study has great potential to be used as a biomarker to detect early signs of sarcopenic back muscle function.

## Introduction

Sarcopenia, the progressive loss of muscle strength and muscle mass with age [1] is defined as a muscle disease rooted in adverse muscle changes that occur across a lifetime [2]. Muscle weakening and atrophy begin in the fourth decade of life with a rate of strength loss of approximately 1% per year, and accelerates in the later decades [3]. To date, a significant body of research has identified structural and morphological alterations in the muscles and the nervous system [4, 5] that are all likely associated with the aging-related decline in neuro-muscular functions, such as muscle weakness and reduced postural performance [6–9]. These age-dependent structural changes are known to be preceded by a loss of preferably highly myelinated axons that are the neuronal components of high threshold motor units (for review see [5, 10]). This axonal apoptosis is accompanied by loss of type II muscle fibers and muscle fiber atrophy but can be functionally well-compensated for decades through neuronal regenerative mechanisms and regular muscle training [4]. By contrast, lack of physical activity, chronic diseases, and malnourishment precipitate the decline in muscle strength and mass that takes place with age [11]. Therefore, the early detection of patients at risk for sarcopenia and the initiation of early interventions in these individuals are of utmost interest in the context of reducing the health burden of sarcopenia in their later life [12–15]. To achieve this goal, as endorsed by the European working group for sarcopenia [2], there is a strong need for function-based biomarkers that: 1) can accurately diagnose the very early forms of sarcopenia in a reliable way, 2) are feasible for the vast majority of older adults, and, 3) are easily performed in the primary care office [16].

Sarcopenia is diagnosed by muscle function and structure related tests, i.e., muscle mass, hand grip strength and gait velocity [1], as well as knee extension strength and power tests [2]. Whereas these tests establish the diagnosis of sarcopenia they seem unsuitable to achieve very early detection of sarcopenia before the related muscle functional impairments occur [17, 18]. Studying the neuromuscular functional decline of the lumbar back extensors muscles would be particularly promising, because these muscles have a higher than average rate of muscle loss with 2.5% per year [19]. In addition, poor back extension strength and endurance was found to be predictive of future falls, impaired mobility, frailty, and the need for institutionalization in older adults [20–23]. Recent observations by this research group found the surface electromyographic (SEMG) based measures of back muscle fatigue capable of identifying early age-dependent changes in individuals who did not demonstrate a major back strength decline with aging [24]. In this study, the fatigue related spectral changes (Median Frequency [MF]-SEMG) recorded during a 30s submaximal sustained back extension at 80% of maximal voluntary effort revealed a significantly smaller spectral compression toward the lower frequencies in older adults as compared to young healthy individuals. During an 80% of maximum isometric contraction all motor units of the back-extensor muscles are assumed to be active. As the fatigue-related changes are preferably mediated by the slowing of the muscle fiber conduction velocity of fast fatiguing, higher threshold motor units, these flatter MF-SEMG slopes in seniors most likely arose from a proportionally smaller number of fast fatiguing muscle fibers [24, 25], indicative of less glycolytic activity in these muscles. Such decline in muscle glycolytic activity likely results from neuronal incomplete regenerative mechanisms following axonal loss of higher threshold motor units with advancing age [8, 9, 26, 27]. This suggests that the MF-SEMG fatigue method has a high potential to detect the precursors of sarcopenia before muscle weakness can occur. However, an 80% maximum voluntary contraction (MVC) isometric trunk extension maintained for 30 s is close to a maximum performance test. Such tests might work well for most active healthy seniors, but could create an increased risk of vertebral fractures in patients with bone diseases of the spine, or may not be feasible in pre-frail persons.

Adopted training protocols that are widely recommended for progressive resistance exercise to novice training seniors [28, 29] would be less demanding and thus allow the monitoring of SEMG back muscle fatigue in a safe way, if SEMG monitored back extensor muscle fatigue could be induced. Such a training protocol would need to comply with the recommended load (exceeding 40% of maximum) to induce EMG fatigue, while both the perceived effort and back extensor fatigue at the end of the exercise would only be moderate. However, the SEMG recorded from cyclic exercises is non-stationary; in this case either traditional Fourier based transformation cannot be applied, or most of the time frequency and wavelet based analyses reveal limitations for the processing of the spectral SEMG components [30–32]. Novel techniques suitable for the spectral analysis of SEMG data collected during dynamic contractions [33, 34] are based on the Cohen-Posch class time-frequency analysis and are the method of choice in estimating the frequency content of a quasi-cyclo-stationary SEMG signal (i.e., signals whose frequency content is modulated in a pseudo-periodic manner superimposed on low-frequency trends). This technique has been successfully administered when analyzing either quadriceps or back muscle fatigue from cyclical exercises in a reliable way (e.g. [33–35]).

Motivated by the goal to develop novel biomarkers that might be used to screen for very early forms of sarcopenia, the primary objective of this research was to examine whether or not age specific effects could be observed in the surface electromyographic representation of healthy individuals engaging in a cyclic back extension exercise. By simultaneously monitoring back muscle activity using these novel SEMG processing techniques, we hypothesize that with the duration of this submaximal cyclic back exercise performed with a load that equated 50% of isometric back extension maximum, the SEMG amplitude would increase to a smaller extent in older than in younger adults. This result is expected because in older adults, the smaller proportion of fast-fatigable motor units requires less fatigue-related compensation; therefore, there would be a smaller increase in net excitatory drive to the alpha motor neuron pool. Likewise, the time-frequency representation of the SEMG data would be less compressed toward the lower frequencies in older as compared to younger individuals, because the proportion of fast-fatiguing muscle fibers is reduced in the muscles of older adults [36]. Due to sex-specific differences in back muscle fiber size [37], we further hypothesized that there are significant differences in the SEMG data characteristics between males and females. Furthermore, the test-retest reliability of these measurements was assessed.

## Material and methods

### Ethics statement

The study protocol was approved by the Ethics Committee of the City of Vienna - Number: “EK 11–064-VK-NZ” Full address: Ethikkommission der Stadt Wien Magistratsabteilung 15, Gesundheitsdienst der Stadt Wien 3., Thomas-Klestil-Platz 8 Town Town Tel.: 01/4000–87,523. All participants provided written informed consent. The data collection was carried out in accordance with the directives of the Declaration of Helsinki. Participants received financial compensation for their participation in the study.

### Participants

Study volunteers were recruited by word of mouth, by presenting the study to groups of older adults attending institutions with a focus on leisure and social activities in and around the city of Vienna, and by posting flyers in appropriate areas at companies near the Karl-Landsteiner Institute of Outpatient Rehabilitation Research where the data collection took place. A total of 86 asymptomatic volunteers (40 females, between 18 and 90 years of age) were enrolled in the study.

Subjects were eligible to participate in the study if: they were generally healthy, i.e., were free of co-morbidities (mild diabetes, well controlled hypertension, mild osteoarthritis of lower limb weight bearing joints were included); had no major general health problems that would interfere with testing; were free from any functional limitations (reported independent walking distance exceeded 800 m, timed up-and-go test less than 10 s, Tandem-stand exceeded 10 s, chair rise test less than 15 s); were independent in their activities of daily life; performed a normal level of physical activity (but did not participate in competitive sports more than two times per week); and, if they were free from any risk factors that could preclude them from participating in vigorous exercise. Physical Medicine & Rehabilitation physicians screened all participants.

Volunteers were not considered eligible to participate in the study if: 1) they were unable to follow instructions given in German; 2) experienced more than five mild back or referring back pain episodes (Visual Analog Scale] > 30) lasting more than two days each within the past year; 3) had a history of spine surgery or any kind of specific diseases of the spine (tumor, inflammation, osteoporosis, fracture); 4) were pregnant; 5) complained about any medical condition that might interfere with maximum strength or submaximal endurance testing; or, 6) had a body mass index (BMI) exceeding 35 kg/m2.

### Experimental protocol

#### Schedule of assessments and tasks

Participants completed a total of three different tasks as follows: 1) Basic anthropometric measurements and questionnaires that assessed their motivation and physical activity level; 2) warm-up and maximum isometric back extension (MVC) tests; 3) 20 min of rest, in which the surface EMG electrodes were attached; 4) one sustained isometric back extension at 80% of maximum for 30s; and, 5) after a resting interval of approximately five minutes, a dynamic fatigue task performed with a load equating to 50% MVC. All tests were guided by three experienced examiners (CS, MW, RH) and a certified clinical psychologist (BP).

The complete set of measurements was repeated in a second experimental session one to two days after the first, and further repeated in a third session six weeks later.

#### Equipment and tests

##### Back extension dynamometer

A back-extension device (F110 extension; DAVID® health solutions, Helsinki, Finland) was used to assess maximum isometric back extension torque. The dynamometer is described elsewhere in detail [38], and consists of a “hip fixation mechanism” that comprises five components: footplates adjustable to lower leg length, knee pads adjustable to thigh length, a pelvic belt, a seat adjustable for height, and a dorsal back pad. Participants were seated on the isometric machines in accordance with the manufacturer’s recommendations, i.e., with the longitudinal axis of their knees parallel to the seat, their trunk flexed forward at 30°, and their arms hanging relaxed at either side of their trunk. Due to the dorsal back pad, undisturbed EMG recording from lumbar extensor muscles is limited with this device.

##### Back extension device that examined static and dynamic muscle fatigue

To allow undisturbed EMG recording from the back-extensor muscles, the sustained 80% MVC, static back extension, and the 50% MVC dynamic exercise were each performed with the “Total Trunk” (TechnoGym®, Italy); “Total Truck” is purely an exercise, as opposed to a strength test, device. This device is constructed similarly to the F110 extension device (DAVID® health solutions, Helsinki, Finland), and also consists of a “hip fixation mechanism” like the F110 device. It is, however, equipped with a dorsal sacral pad instead of a back pad. As with the DAVID device, the device has footplates adjustable to the lower leg length, knee pads adjustable to the thigh length, a pelvic belt, and a seat adjustable for height.

##### Hand grip strength testing

Patients were seated in an upright position without back support, both feet on the floor, and with both elbows flexed at 90 degrees and the wrists in neutral position according to the manufacturer’s instructions. They then performed a series of three maximum grip strength tests (Jamar; USA), where the order was alternated between the right and left hand (each test was followed by rest interval of 20 s). If the best two tests varied by more than 10%, a further test was performed so that a consistent maximum could be achieved. All values were monitored and recorded.

##### Surface EMG

Electromyographic signals were monitored using active double parallel-bar electrode sensors that also integrated triaxial accelerometric sensors (Model Trigno, DelSys®, Boston, MA, USA). After the skin had been abraded with alcohol and, if necessary, shaved, electrodes were positioned bilaterally over the multifidus muscle at L5, the longissimus at L2, and the iliocostalis lumborum muscles at L1, considering muscle fiber direction and the positioning recommended by the SENIAM project [39] and by previous studies [40]. All sensors were attached to the skin by a double-sided adhesive interface and testers were well trained in administering the sensors. Landmark locations served to assign validity of the SEMG signal because it is difficult to capture the multifidus muscle with surface electrodes. The SEMG signals were acquired at a total effective gain of 909 V/V ± 5%, a bandwidth of 20-450 Hz and a baseline noise < 0.75 μV (RMS). The SEMG signals were sampled at 2000 Hz using a 16-bit analog/digital (A/D) board and EMG works acquisition software (DelSys®, Inc., Boston, MA, USA).

##### Accelerometric signal

A triaxial accelerometric sensor integrated into the double parallel-bar EMG electrode sensor (Trigno, DelSys Inc.®, Boston, MA, USA) was attached to the lever arm of the dynamometer in a standardized way. It monitored the direction of the trunk movement. By considering the time elapsed for each cycle, mechanic variables like the range of trunk movement, peak, and average movement velocity of the concentric and eccentric bouts of the exercise could be monitored. The triaxial accelerometers acquired pre-amplified signals with a dynamic range of ±1.5 g, a maximum resolution of 0.016 g/bit, and a bandwidth of dc-50 Hz. The accelerometer signals were sampled at 160 Hz with a resolution of 8 bits and EMG Works® acquisition software.

##### Questionnaires

Participants completed the International Physical Activity Questionnaire (IPAQ) on each test day [41]. At the end of the 50% MVC cyclic trunk extension exercise subjects rated their back-muscle fatigue level on a Borg scale, which has anchors at 0 (no fatigue at all) and 10 (most severe fatigue ever experienced).

#### Test procedures

##### Maximum back extension test

After the participants were securely positioned in the F110 device (DAVID®) following the manufacturer’s recommendations, they performed a warm-up at low loads to familiarize themselves with the equipment and test procedures. Thereafter, participants completed two consecutive maximum isometric contractions under supervision. If the second test varied by more than 10%, or if the moment of peak effort was achieved later than 3 s after the onset of the contraction, further trials were permitted until a consistent maximum was achieved. The best score out of two consistent trials was recorded and stored. Verbal instructions and encouragement provided by the testers were standardized and regularly supervised by a clinical psychologist (BP).

##### 80% back extension test

After the electrodes were attached to the muscles of interest and checked for function, participants were seated on the Total Trunk device using the same positioning variables that were used for the DAVID® device. Participants maintained a 30° ante flexed trunk position for at least 30s with a load equal to 80% MVC that had been derived from the best maximum trunk extension moment (Newton meters, Nm) with the F110 DAVID® device. Load was calculated by the mathematical product of the moment recorded by the load cell of the dynamometer and the moment arm defined by the distance of the back restraint and the load cell.

##### Cyclic back extension exercise

Participants performed the cyclic back extension tests in a sitting position with their arms hanging relaxed at either side of their trunk. In a practicing session without external load, participants repetitively flexed and re-extended their trunks between the upright sitting position and 40° forward flexed position following the pace of a metronome and verbal instructions. Participants were also instructed and briefly trained in a standardized breathing technique with inhalation during the eccentric phase and expiration during the concentric phase of the cyclic exercise. Thereafter, participants were required to perform a total of 25 cyclic contractions with the lever arm loaded equal to the 50% MVC. The movement velocity of the flexion-extension cycle was paced with the metronome set at 2 s intervals (2 s extension and 2 s flexion). The qualitative performance of the dynamic contractions was supervised by experienced testers. If the limit of 25 repetitions could not be reached, or the homogeneity of task performance decreased in range of motion (ROM) or movement velocity, the test was stopped early. If the number of successfully performed cycles exceeded 15 repetitions, it was considered for further data processing.

##### Subjective rating of muscle fatigue

Immediately after the dynamic test, participants were asked to rate their back-muscle fatigue level on a BORG visual analogue rating scale. The anchor points of the scale were zero indicating “no fatigue at all” and 10 indicating “the most severe fatigue ever experienced”.

### Signal processing

MATLAB routines (The MathWorks, Inc., Natick, MA, USA) were used for EMG data processing. All data was preprocessed by skipping the first three seconds of the static task and the first eight seconds (first two cycles) of the dynamic task, and by removing artifacts. EMG data was filtered using a 20 Hz high-pass and 500 Hz low-pass Butterworth filter design. RMS-EMG calculations were performed from the second to the fifth second of the sustained 80% MVC contraction, as described previously [24]. These values were used for normalization of the RMS-EMG values recorded from the dynamic contractions.

**Fig. 1:**
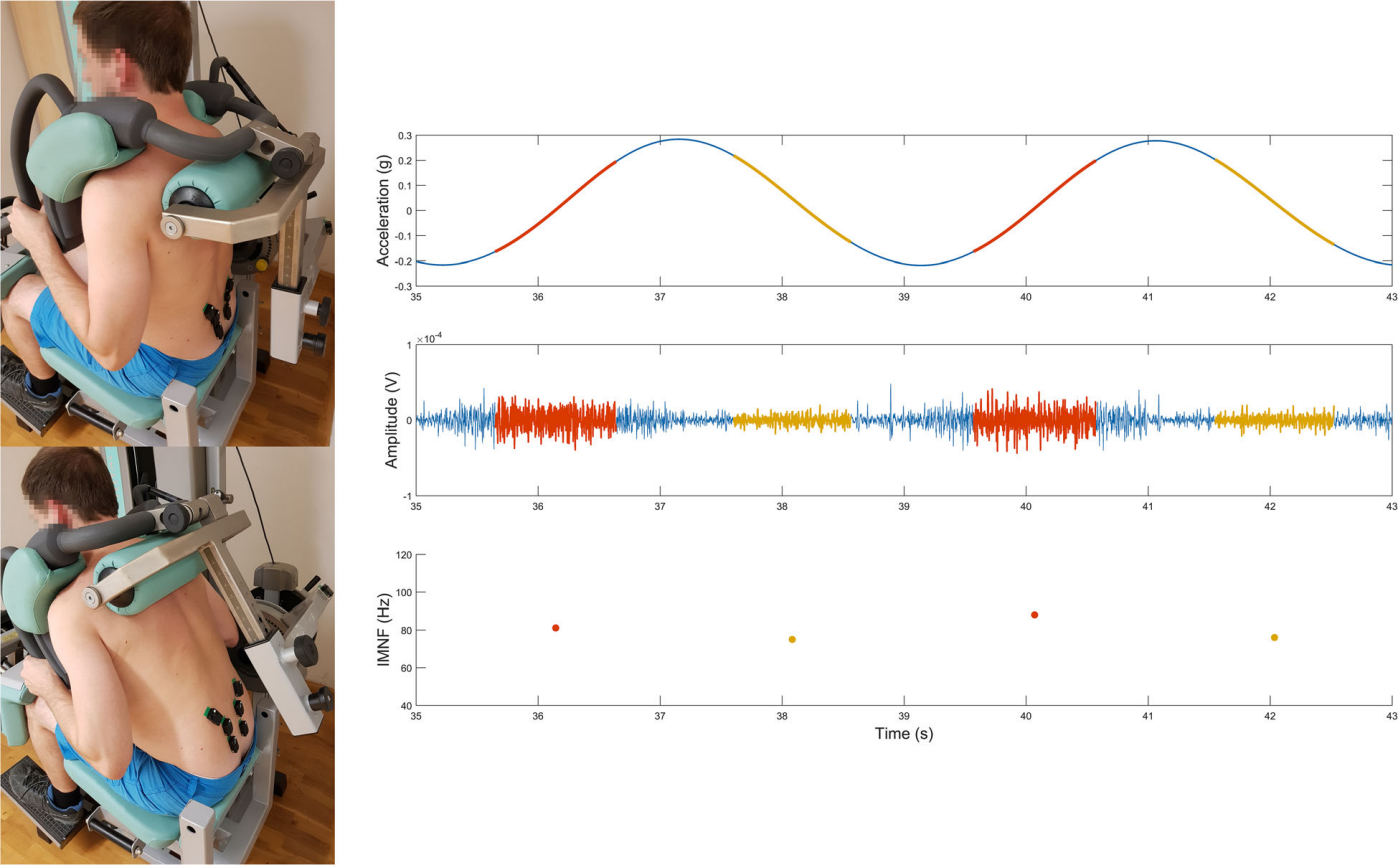
The left part of this figure illustrates the positioning of the SEMG sensors during the test as well as the dynamic exercise in extension (top left) and flexion (bottom left). The right part of this figure illustrates the trunk angular displacement (top), the angular velocity (second row), the raw SEMG data (third row), and IMDF-SEMG estimates (bottom) over the duration of two trunk exercise cycles for concentric (highlighted in red) and eccentric (yellow) phases. Data is shown for a representative subject

EMG signals of the dynamic fatigue task were sequenced according to their concentric and eccentric phases using acceleration data from one electrode positioned on the lever arm of the Total Trunk device. These on-average 1 s EMG intervals corresponded in equal portions to trunk ROM segments during flexion and extension to avoid acceleration, deceleration, and turning phases during the exercise. Thus, movement velocity and muscle length - torque geometry were comparable in the EMG representations not only between the shorting and lengthening of the cyclic exercise but also between the individuals tested (see Fig. 1).

Root mean square (RMS) and instantaneous median frequency (IMDF) SEMG variables were computed for all sequenced SEMG intervals recorded during the dynamic shortening and lengthening contractions. SEMG sections, consisting of approximately 2000 samples, were split up into windows of 500 samples. These windows were overlapping by 50%, whereas IMDF and RMS calculations of the first and last 125 samples of the approximately 1 s intervals were rejected to ensure the extraction of the central portion of the angular range of motion. IMDF calculations are based on Cohen Class Time-Frequency representations [42] for computing instantaneous frequency distributions. Our approach is based on findings from comparisons of five different time-frequency transformations, namely, the Wigner-Ville, the smoothed Wigner-Ville, the Cone Kernel, the reduced interference, and the Choi-Williams. These techniques were applied to computer-synthesized realizations of stochastic processes, as well as to real signals detected during different types of contractions in healthy human volunteers [30]. Cohen Posch representations were obtained by first computing a Cohen Class [30] time-frequency representation of the EMG data and then applying an iterative algorithm to remove undesirable oscillating terms that mark Cohen Class representations [43]. Cohen Class transformations may be seen as an extension of the correlogram technique [30]. The autocorrelation function is replaced by the instantaneous autocorrelation function [42]. Its Fourier transformation is time-dependent and constitutes a representation of the instantaneous frequency content of the EMG signal [30]. As Cohen Class representations provide a relatively unstable estimate of the EMG frequency content, Cohen-Posch representations [44] were derived from them via iterative adjustments of the time and frequency marginals [43]. The IMDF-EMG value was calculated for one portion of each phase of the exercise cycle, across all the cycles of the exercise [33].

The electrical manifestations of muscle fatigue were assessed for each series of contractions by computing the least squares regression line through the 25 IMDF-estimates relative to the 25 signal bursts of each contraction series. The corresponding IMDF SEMG values of one exercise session were averaged to obtain one IMDF value per cycle and phase. Figure 1 shows the procedure described above applied to the signal collected from the multifidus muscle of a subject during a series of back flexion-extension cyclical movements (legend to Fig. 1). The RMS-SEMG values were normalized by the RMS-SEMG obtained during 4 s (i.e. seconds 3 to 7) of the 80% static, sustained contractions.

Linear regression was applied to the time series of SEMG values to calculate the rate of change in RMS-SEMG and IMDF-SEMG over time; these measures are indicative of muscle fatigue. Following previous recommendations [45], both the RMS and the IMDF slopes were divided by their corresponding intercept values to achieve normalized SEMG indices of muscle fatigue, which accounted for differences in subcutaneous tissue thickness amongst test participants. In order to improve reliability, the scores of each of the three electrode pairs at the three lumbar levels (L1, L2, and L5) were averaged to increase their reliability [46].

#### Accelerometric signal analysis

Trunk range of motion was monitored via the sagittal angular displacement as calculated by a geometrical procedure using direction of gravity as reference; this process has previously been described in detail [47]. To calculate individual angles, the acceleration data of the electrodes from every position was used.

### Definition of variables

The following dependent variables were used in the analysis: MVC as a measure of trunk extensor strength, mean RMS-SEMG and IMDF-SEMG values derived from the initial values of linear regression models, mean RMS-SEMG and IMDF-SEMG slopes normalized by the RMS-SEMG, and IMDF-SEMG initial values. The independent variables were subjects’ age (older or younger than 50 years of age) and sex. All statistical analyses were done in the R environment for statistical computing [48].

### Age and sex specific subgroups

**Fig. 2:**
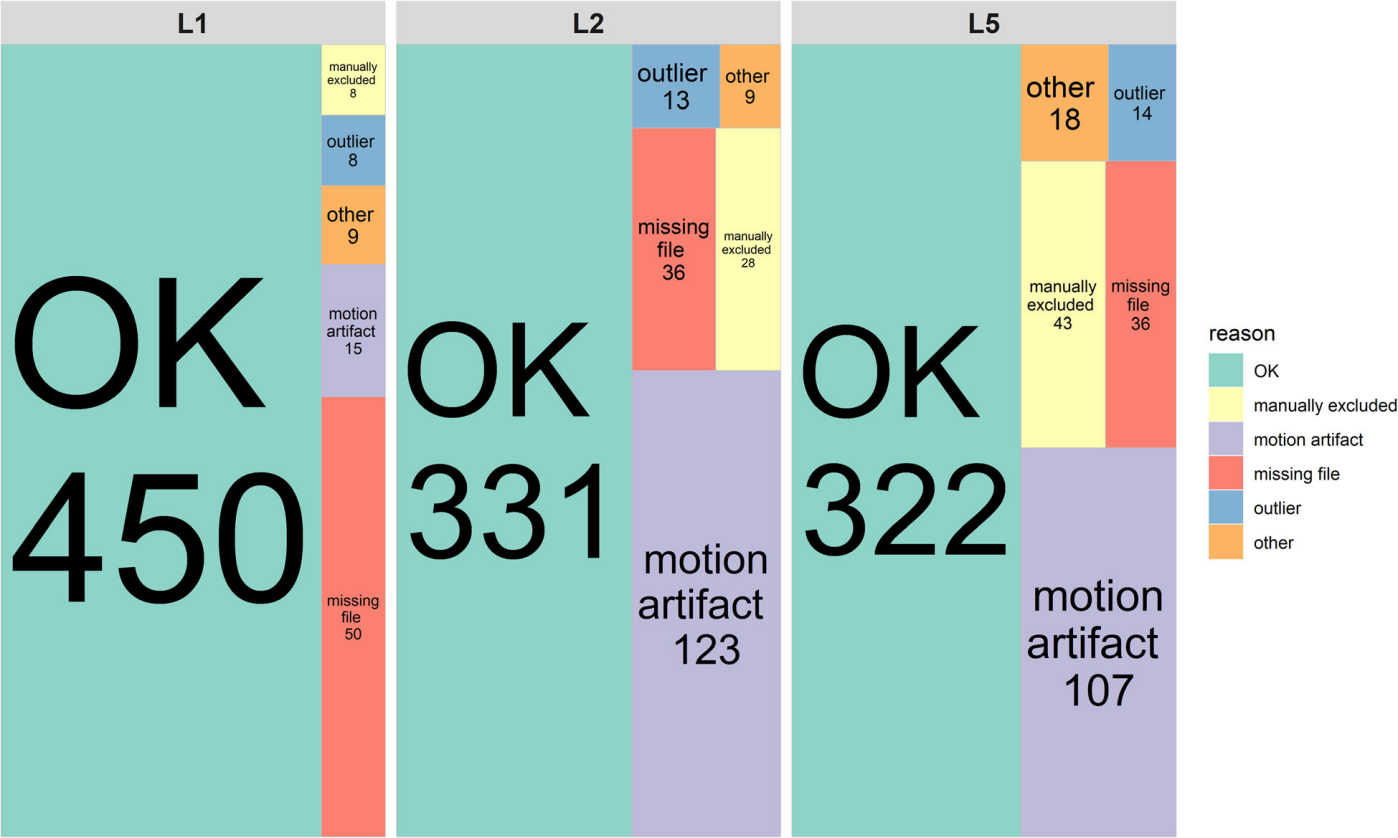
shows the main reasons for not further processing the SEMG signal. Note that the EMG recordings were from three pairs of electrodes (L5 = multifidus, L2 = longissimus, L1 = iliocostalis lumborum) and the experiments were performed on three different days

For the analysis of age and sex specific differences, biomechanically comparable (velocity and acceleration) dynamic tasks and artefact-free SEMG data from the full set of electrodes was available from 27 older and 29 younger participants (Fig. 2). This was done to control for any confounders that could occur from group imbalances in the biomechanics of task performance or due to missing EMG variables. Our primary analysis was performed with fitted data of missing SEMG variables of individual electrodes. Fitting of data was performed if the SEMG recordings of one of the two (paired) electrodes per segment were undisturbed. Consequently, the undisturbed SEMG was duplicated and onset and slopes values were corrected by the left-right imbalance ratios of the respective lumbar segment obtained from the 56 individuals with the full data set. Consequently, data from a total of 35 older and 41 younger individuals were available for analysis. A secondary analysis was performed with the full set of electrodes available. Results of the secondary analysis are not shown but can be provided upon request.

Descriptive statistics served to summarize the participants’ characteristics. To evaluate whether maximum back extension moment or SEMG fatigue variables differed between the two age-specific and sex-specific groups, mixed effects model with “age”, “sex” and “test number” as fixed factors and the random factor “persons” were computed for each outcome variable. *P*-values were Bonferroni corrected (five electrode levels) and considered significant if *p* ≤ 0.01. The sample size estimate for the mixed ANOVAs was calculated with Monte Carlo power simulation [49] and was based on findings from two previous studies [24, 50]. Considering a total of five comparisons at an alpha of 0.01 and a power of 0.9 (1-beta), a sample size of 80 participants was necessary to detect medium age- or sex-specific differences between groups.

### Reliability of EMG fatigue measurements

Generalizability Theory (G-Theory) [51–53] was used to examine reliability of the SEMG measures. As compared to classical test theories, the G-theory takes into account how the consistency of outcomes may change if a measure is used to make absolute (criterion referenced) versus relative (norm referenced) decisions. Using a “multi-factorial random-effects Analysis of Variance (ANOVA)” model that included several sources of measurement error related to: subject, day, side, subject x day, and subject x side, the absolute standard error of measurement (SEM) values as well as the respective coefficients of dependability (D), the latter is a type of Intra Class Correlation Coefficient (ICC), were calculated. Note that absolute decisions are criterion-referenced and occur if the subject’s measurement results are independent of the performance of other subjects.

## Results

Of the 87 participants tested, a total of 56 participants had a fully artefact free EMG data set that included all six electrode-recording sites and was comparable in the biomechanics when performing the task. Of the 31 participants excluded from the analysis, in most of the cases the SEMG signal taken from one side of the pairwise electrodes was contaminated with strong artefacts, usually due to a slight and intermittent electrode detachment during performing the cyclic exercise. Biomechanical inconsistencies due to significant changes in task performance or the early cessation of the fatigue test occurred in three cases, and reasons for exclusion of individual EMG signal recordings are provided in Fig. 2. For the analyses of the fitted EMG data, a total of 76 individuals could be considered.

Participants’ demographic variables “age”, “height”, and “weight”, their functional variables “maximum back extension strength”, “hand grip strength”, and their fatigue ratings at the end of the cyclical exercise did not differ from those individuals who had to be excluded from further analysis (Table 1).

**Table 1:** Demographic and variables related to the biomechanics of test performance. *P*-values are provided for differences between age groups and for differences between included (*n* = 76) and excluded (*n* = 20) subjects

	Younger adults Mean (SE)	Older adults Mean (SE)	Younger vs. older adults *	Included vs. excluded *
N	41	35		
Age (years)	32.8 (2.6)	67.0 (1.7)	< 0.001	0.8
Height (cm)	173.8 (1.6)	168.4 (2.2)	0.054	0.5
Weight (kg)	73.3 (2.3)	72.0 (3.0)	0.7	0.7
BMI (kg/m^2^)	24.3 (0.5)	25.3 (0.6)	0.2	0.6
Back Extension Strength (Nm)	241.4 (15.3)	207.9 (14.5)	0.1	0.4
Grip Strength (kg)	39.6 (2.7)	32.3 (3.4)	0.1	0.7
Fatigue (BORG Scale 0–10)	5.1 (0.5)	3.6 (0.4)	0.028	0.9
IPAQ TPA (MET/week)	5234 (1133)	10,701 (2425)	0.038	0.7
EMG Velocity ex (rad/s)	−0.48 (0.02)	−0.44 (0.02)	0.2	0.5
EMG Velocity con (rad/s)	0.57 (0.02)	0.58 (0.02)	0.6	0.9
EMG ROM (rad)	0.55 (0.02)	0.54 (0.02)	0.5	0.9

*p-value *TPA* Total Physical Activity, *ROM* Range of Motion

When comparing groups for age specific differences neither maximum back extension strength nor maximum hand grip strength differed significantly. Participants’ total physical activity scores (TPA) were significantly higher in the older than the younger individuals, and their back-muscle fatigue ratings indicated significantly more fatigue at the end of the cyclic exercise in the younger than older participants (Table 1).

Of the biomechanical performance variables of the cyclic back extension exercise, the trunk range of motion as well as movement velocity calculated for the concentric and eccentric phases separately were comparable between participants of the two age groups. In addition, these mechanic variables remained constant throughout the dynamic exercise and in each of the two age specific subgroups (Table 1).

### Age dependent RMS-SEMG representations accompanying the cyclic exercise

The normalized RMS-SEMG onsets derived both from the concentric and the eccentric phases of the cyclic exercise did not significantly differ between younger and older individuals, nor between males and females. It is worthwhile to note that the initial RMS-SEMG values would be approximately twofold in size if the concentric portions of the exercise were compared with the eccentric ones.

If the concentric portions of the exercise were considered, the RMS-SEMG of all muscles, the longissimus and iliocostalis lumborum recording sites, significantly increased in individuals of both age and sex groups over the course of the exercise routine and did not significantly differ between younger and older participants, nor between males and females. As opposed to the younger participants, no significant RMS-SEMG fatigue increases were observed for the multifidus muscle of the older participants but differences between age groups did not reach a level of significance. For the eccentric portions of the exercise, the RMS-SEMG slopes were found to significantly increase in younger participants in the iliocostalis lumborum electrode recording site. However, neither the respective age nor sex specific differences reached a level of significance. Interestingly, those electrode sites that revealed the most negative IMDF slope showed a significant decrease in the normalized RMS-SEMG changes for the eccentric portions of the exercise, but did not differ significantly between age and sex groups. Tables 2 and 3 display the median RMS-SEMG onsets and changes over time normalized by the RMS-SEMG values derived from the 80% isometric MVC test. Our second analysis with the full, unfitted data set revealed similar findings.

**Table 2:** Results of the concentric RMS-EMG obtained from 76 healthy individuals that performed the cyclic submaximum lifting task. For these 76 subjects a fitted EMG dataset was available on at least one test day and the mechanical variables of test performance were comparable between age groups. Note that significant changes of the RMS-SEMG with the duration of the exercise and normalized to the onset are indicated by “*” (Bonferroni corrected sig. Level = *p* < 0.005)

*n* = 153		Intercept					Slope normalized to intercept				
Level		Mean (SE)	Age F; p	Sex F; p	Age:Sex F; p	tnr F; p	Mean (SE)	Age F; p	Sex F; p	Age:Sex F; p	tnr F; p
All	< 50	1.31 (0.04)	0.3; 0.6	1.8; 0.2	2.7; 0.1	2.3; 0.1	0.32 (0.06)*	1.7; 0.2	0.7; 0.4	1.4; 0.2	0.6; 0.6
	> 50	1.28 (0.04)					0.18 (0.04)*				
multifidus (L5)	< 50	1.36 (0.07)	1.0; 0.3	4.3; 0.043	2.3; 0.1	1.2; 0.3	0.34 (0.08)*	2.2; 0.1	0.0; 0.9	1.8; 0.2	0.8; 0.5
	> 50	1.27 (0.04)					0.12 (0.04)				
longissimus (L2)	< 50	1.41 (0.05)	1.2; 0.3	1.6; 0.2	1.3; 0.3	1.6; 0.2	0.29 (0.06)*	0.3; 0.6	1.0; 0.3	0.4; 0.5	0.0; 1.0
	> 50	1.32 (0.05)					0.23 (0.05)*				
iliocostalis lumborum (L1)	< 50	1.16 (0.04)	1.3; 0.3	0.1; 0.7	1.6; 0.2	0.6; 0.6	0.33 (0.05)*	2.3; 0.1	2.7; 0.1	1.4; 0.2	1.2; 0.3
	> 50	1.25 (0.04)					0.20 (0.04)*				
most negative electrode	< 50	0.95 (0.03)	1.4; 0.2	0.0; 0.9	1.1; 0.3	0.7; 0.5	−0.04 (0.05)	0.1; 0.7	0.1; 0.7	0.9; 0.4	0.0; 1.0
	> 50	1.02 (0.03)					−0.02 (0.03)				
uncomp. Imbalances	< 50						26.4 (3.0)	0.1; 0.7	3.0; 0.1	0.4; 0.5	0.6; 0.5
	< 50						24.6 (1.9)				
comp. Imbalances	< 50						−3.1 (3.5)	0.9; 0.3	0.2; 0.7	0.0; 0.8	1.7; 0.2
	< 50						−8.7 (2.9)				

*uncomp*. uncompensated, *comp*. Compensated, *tnr* Test day, *p p*-value, *F F*-value * = *p* < 0.005

**Table 3:** Results of the eccentric RMS-EMG obtained from 76 healthy individuals that performed the cyclic submaximum lifting task. For these 76 subjects a fitted EMG dataset was available on at least one test day and the mechanical variables of test performance were comparable between age groups. Note that significant changes of the RMS-SEMG with the duration of the exercise and normalized to the onset are indicated by “*” (Bonferroni corrected sig. Level = *p* < 0.005)

*n* = 153		Intercept					Slope normalized to intercept				
Level		Mean (SE)	Age F; p	Sex F; p	Age:Sex F; p	tnr F; p	Mean (SE)	Age F; p	Sex F; p	Age:Sex F; p	tnr F; p
All	< 50	0.69 (0.02)	0.1; 0.7	1.3; 0.3	2.1; 0.2	0.7; 0.4	0.35 (0.12)	2.5; 0.1	1.0; 0.3	0.8; 0.4	0.0; 1.0
	> 50	0.71 (0.02)					0.01 (0.02)				
multifidus (L5)	< 50	0.75 (0.05)	0.3; 0.6	1.7; 0.2	0.4; 0.5	0.2; 0.8	0.53 (0.23)	2.1; 0.2	0.5; 0.5	0.8; 0.4	0.2; 0.8
	> 50	0.72 (0.03)					−0.04 (0.02)				
longissimus (L2)	< 50	0.71 (0.03)	0.0; 0.9	0.9; 0.3	2.6; 0.1	0.32; 0.7	0.26 (0.12)	1.0; 0.3	0.8; 0.4	0.0; 0.8	0.8; 0.4
	> 50	0.71 (0.03)					0.06 (0.03)				
iliocostalis lumborum (L1)	< 50	0.61 (0.02)	5.9; 0.018	0.1; 0.8	1.9; 0.2	0.93; 0.4	0.27 (0.07)*	4.4; 0.039	1.5; 0.2	2.0; 0.2	0.5; 0.6
	> 50	0.69 (0.02)					0.01 (0.03)				
most negative electrode	< 50	0.48 (0.02)	6.5; 0.013	0.1; 0.8	4.2; 0.045	0.85; 0.4	−0.19 (0.07)*	0.2; 0.6	0.0; 1.0	0.3; 0.6	0.4; 0.7
	> 50	0.56 (0.02)					−0.21 (0.02)*				
uncomp. Imbalances	< 50						26.9 (3.3)	0.8; 0.4	1.9; 0.2	0.1; 0.8	0.5; 0.6
	< 50						22.9 (1.9)				
comp. Imbalances	< 50						−0.3 (4.0)	2.0; 0.2	0.9; 0.4	0.4; 0.5	0.7; 0.5
	< 50						−8.2 (2.7)				

*uncomp*. Uncompensated, *comp*. Compensated, *tnr* Test day, *p p*-value, *F F*-value * = *p* < 0.005 (indicates significant RMS-SEMG fatigue changes)

### Age dependent IMDF SEMG representations accompanying the cyclic exercise

The IMDF-EMG onsets obtained from the cyclic back exercise tended to be slightly higher in older as compared to younger individuals. Neither age-specific nor sex-specific differences reached a level of significance for both the concentric and eccentric contraction phases.

The IMDF-SEMG slopes normalized to the IMDF onset values significantly decreased with the duration of the exercise. Although these slopes were generally more pronounced in younger than in older participants, significant age specific differences were predominantly observed when the IMDF-SEMG slopes were derived from the concentric phases of the exercise. This was true for all electrodes, the pair of electrodes with the most negative slope, and the multifidus and longissimus (in a tendency significant) electrode recording sites. For the eccentric phases of the cyclic exercise, significant age specific differences were observed for the multifidus muscle and the electrode with the steepest IMDF-SEMG slope. No sex specific differences were observed, except for the most negative electrode recording site. There were neither age nor sex specific differences observed for the imbalance scores. The results of the age and sex specific differences of the IMDF-EMG are presented in Table 4 for the concentric phase and in Table 5 for the eccentric phase. Our secondary analysis including the full, unfitted data set revealed age specific differences for the IMDF onsets and slopes that were similar to those obtained from our primary analysis.

**Table 4:** Results of the concentric IMDF-EMG obtained from 76 healthy individuals that performed the cyclic submaximum lifting task. For these 76 subjects a fitted EMG dataset was available on at least one test day and the mechanical variables of test performance were comparable between age groups. Note that significant changes of the IMDF-SEMG with the duration of the exercise and normalized to the onset are indicated by “*” (Bonferroni corrected sig. Level = *p* < 0.005); significant groups effects are Bonferroni corrected and given in boldface if *p*< 0.01

*n* = 156		Intercept					slope normalized to intercept				
Level		Mean (SE)	Age F; p	Sex F; p	Age:Sex F; p	tnr F; p	Mean (SE)	Age F; p	Sex F; p	Age:Sex F; p	tnr F; p
All	< 50	59.9 (1.2)	1.6; 0.2	0.1; 0.7	0.6; 0.4	0.3; 0.8	− 0.16 (0.02)*	**10.0; 0.002**	1.7; 0.2	0.0; 0.9	1.5; 0.2
	> 50	61.9 (1.2)					−0.07 (0.01)*				
multifidus (L5)	< 50	67.5 (1.7)	4.4; 0.04	0.7; 0.4	0.1; 0.7	1.3; 0.3	−0.25 (0.02)*	**14.2; < 0.001**	1.4; 0.2	0.0; 1.0	0.8; 0.4
	> 50	73.5 (1.7)					−0.10 (0.02)*				
longissimus (L2)	< 50	58.9 (1.6)	1.1; 0.3	0.2; 0.7	0.8; 0.4	0.5; 0.6	−0.18 (0.02)*	6.5; 0.013	2.7; 0.1	0.0; 0.9	1.2; 0.3
	> 50	60.9 (1.4)					−0.08 (0.02)*				
iliocostalis lumborum (L1)	< 50	53.2 (1.1)	0.4; 0.5	0.4; 0.5	0.5; 0.5	1.0; 0.4	−0.06 (0.02)*	1.5; 0.2	0.0; 0.9	0.4; 0.5	2.8; 0.06
	> 50	51.4 (0.9)					−0.03 (0.01)*				
most negative electrode	< 50	47.5 (0.9)	1.4; 0.2	0.0; 0.9	0.6; 0.4	0.6; 0.5	−0.34 (0.02)*	**17.5; < 0.001**	**8.2; 0.006**	0.0; 0.9	0.5; 0.6
	> 50	48.6 (0.9)					−0.20 (0.02)*				
uncomp. Imbalances	< 50						9.5 (0.6)	1.1; 0.3	0.1; 0.8	1.2; 0.3	0.6; 0.6
	< 50						8.6 (0.5)				
comp. Imbalances	< 50						−2.6 (0.8)	0.1; 0.8	4.6; 0.035	0.0; 1.0	0.1; 0.9
	< 50						−2.9 (0.9)				

*uncomp*. Uncompensated, *comp*. Compensated, *tnr* Test day, *p p*-value, *F F*-value * = *p* < 0.005

**Table 5:** Results of the eccentric IMDF-EMG obtained from 76 healthy individuals that performed the cyclic submaximum lifting task. For these 76 subjects a fitted EMG dataset was available on at least one test day and the mechanical variables of test performance were comparable between age groups. Note that significant changes of the IMDF-SEMG with the duration of the exercise and normalized to the onset are indicated by “*” (Bonferroni corrected sig. Level = *p* < 0.005); significant group effects are Bonferroni corrected and are given in baldface if *p*<0.01

*n* = 156		Intercept					Slope normalized to intercept				
Level		Mean (SE)	Age F; p	Sex F; p	Age:Sex F; p	tnr F; p	Mean (SE)	Age F; p	Sex F; p	Age:Sex F; p	tnr F; p
All	< 50	62.36 (1.25)	1.1; 0.3	0.7; 0.4	2.8; 0.1	0.0; 1.0	−0.15 (0.02)*	4.3; 0.043	2.5; 0.1	0.0; 0.9	0.1; 0.9
	> 50	64.41 (1.15)					−0.09 (0.01)*				
multifidus (L5)	< 50	70.97 (1.85)	2.2; 0.1	0.1; 0.8	0.7; 0.4	0.1; 0.9	−0.25 (0.03)*	**11.7; 0.001**	1.7; 0.2	0.4; 0.6	0.5; 0.6
	> 50	76.08 (1.68)					−0.11 (0.02)*				
longissimus (L2)	< 50	61.14 (1.68)	1.5; 0.2	0.1; 0.8	3.0; 0.09	0.6; 0.5	−0.13 (0.03)*	0.4; 0.5	2.0; 0.2	0.0; 0.9	0.1; 0.9
	> 50	63.92 (1.39)					−0.10 (0.02)*				
iliocostalis lumborum (L1)	< 50	54.97 (1.11)	0.5; 0.5	3.5; 0.06	2.3; 0.1	0.6; 0.5	−0.08 (0.02)*	1.1; 0.3	0.3; 0.6	0.5; 0.5	0.3; 0.8
	> 50	53.22 (1.00)					−0.05 (0.01)*				
most negative electrode	< 50	47.47 (0.94)	2.2; 0.1	2.6; 0.1	2.7; 0.1	0.3; 0.8	−0.37 (0.02)*	**12.8; 0.001**	**7.1; 0.009**	0.0; 0.9	0.6; 0.6
	> 50	49.43 (0.93)					−0.25 (0.02)*				
uncomp. Imbalances	< 50						10.1 (0.6)	1.7; 0.2	1.5; 0.2	0.0; 1.0	0.0; 1.0
	< 50						9.0 (0.5)				
comp. Imbalances	< 50						−1.0 (1.0)	0.7; 0.4	4.3; 0.042	0.1; 0.8	1.2; 0.3
	< 50						−2.7 (0.9)				

*uncomp*. uncompensated, *comp*. compensated, *tnr* test day, *p p*-value, *F F*-value * = *p* < 0.005

### Retest reliability of the RMS- and IMDF-EMG representations in older and younger individuals

#### RMS-SEMG

The absolute SEM values of the normalized RMS-SEMG onsets calculated for either the different (bilateral) recording sites separately, for the most negative electrode, or for all electrodes were found to be generally larger for the concentric than the eccentric phases of the exercise. The highest SEM-values were found in the longissimus and iliocostalis lumborum recording sites and lowest ones if all electrode recording sites were considered. By contrast, the absolute SEM values of the normalized RMS-SEMG slopes were generally smaller for the concentric than for the eccentric phases of the exercise. The highest value was found for the longissimus, and the smallest one when all the electrodes were considered. There were no major differences between the two age specific subgroups.

The D-coefficients (a type of ICC) of the initial RMS-SEMG were found to be slightly higher for the concentric phase of the exercise; they ranged between 0.63 (multifidus eccentric) and 0.9 (all electrodes, concentric). The respective D-values of the normalized RMS-EMG slopes were again found to be slightly higher for the concentric than the eccentric phases of the contraction with the lowest value observed from the longissimus (eccentric), and the highest value occurred when all the electrodes were considered (concentric). There were no major age specific differences found for these variables. (Table 6).

**Table 6:** Test-retest reliability of the main outcome variables derived from the RMS-EMG and IMDF-EMG recorded on three different test days

	IMDF								RMS							
	Intercept				Slope normalized to Intercept				Intercept				Slope normalized to Intercept			
	Eccentric		Concentric		Eccentric		Concentric		Eccentric		Concentric		Eccentric		Concentric	
	D^a^	SEM	D^a^	SEM	D^a^	SEM	D^a^	SEM	D^a^	SEM	D^a^	SEM	D^a^	SEM	D^a^	SEM
All electrodes	0.80	4.28	0.83	3.91	0.81	0.05	0.79	0.04	0.87	0.05	0.90	0.09	0.87	1.24	0.90	0.12
< 50	0.83	4.08	0.86	3.58	0.79	0.06	0.74	0.05	0.89	0.05	0.90	0.10	0.86	1.72	0.87	0.14
> 50	0.74	4.53	0.78	4.35	0.76	0.04	0.79	0.03	0.81	0.04	0.89	0.08	0.83	0.07	0.93	0.07
Multifidus (L5)	0.85	4.96	0.87	4.53	0.73	0.08	0.83	0.06	0.63	0.09	0.78	0.13	0.73	3.67	0.76	0.21
< 50	0.84	5.50	0.86	4.81	0.63	0.09	0.85	0.06	0.63	0.10	0.80	0.14	0.73	4.97	0.73	0.27
> 50	0.80	5.28	0.84	5.06	0.71	0.08	0.70	0.07	0.53	0.07	0.68	0.12	0.65	0.09	0.78	0.13
Longissimus (L2)	0.89	4.09	0.91	3.46	0.63	0.09	0.73	0.06	0.69	0.08	0.76	0.15	0.46	0.76	0.80	0.19
< 50	0.90	4.50	0.91	3.79	0.68	0.09	0.71	0.07	0.71	0.10	0.74	0.19	0.40	1.15	0.74	0.25
> 50	0.88	3.70	0.90	3.26	0.45	0.08	0.70	0.05	0.59	0.07	0.71	0.13	0.58	0.17	0.84	0.13
Iliocostalis lumb. (L1)	0.91	2.55	0.90	2.56	0.71	0.05	0.56	0.05	0.82	0.06	0.84	0.13	0.76	0.29	0.81	0.15
< 50	0.91	2.94	0.89	2.98	0.74	0.06	0.49	0.06	0.84	0.07	0.83	0.15	0.70	0.43	0.69	0.22
> 50	0.91	2.10	0.91	2.03	0.61	0.04	0.66	0.04	0.68	0.06	0.81	0.11	0.73	0.09	0.90	0.09
Most negative electrode	0.86	2.90	0.89	2.58	0.81	0.06	0.73	0.05	0.83	0.06	0.84	0.11	0.74	0.28	0.81	0.14
< 50	0.85	3.32	0.89	2.96	0.78	0.06	0.64	0.06	0.85	0.06	0.86	0.12	0.74	0.38	0.79	0.17
> 50	0.89	2.33	0.90	2.08	0.76	0.05	0.73	0.04	0.75	0.05	0.78	0.11	0.74	0.09	0.83	0.09

^a^D = D-value

#### IMDF-SEMG

The absolute SEM values of the IMDF-EMG onsets were similar for the concentric and the eccentric phases of the exercise and revealed the highest value in the iliocostalis lumborum (eccentric phase), and the lowest value was found when all electrodes were considered (eccentric). By contrast, the absolute SEM values of the normalized IMDF-SEMG slopes were found to be slightly smaller during the concentric phases of the exercise. The largest value was found for the multifidus (concentric phase), and the smallest one for the iliocostalis lumborum (concentric phase). There were no major differences between the two age specific subgroups.

D-values for the IMDF-EMG onsets observed at all the different recording sites exceeded values of 0.8 in both the concentric and eccentric contraction phases, indicating excellent reliability. The respective D-values of the normalized IMDF-EMG slopes were similar amongst the two contraction phases and age groups, with the lowest values observed for the iliocostalis lumborum (0.56; concentric phase) and the highest ones for the multifidus (0.84; concentric phase), respectively. Such D-values were similar amongst younger and older individuals except for the longissimus (eccentric phase), where younger participants displayed clearly better values than older ones. All the ICC and SEM values calculated for the different EMG recording sites are provided in Table 4.

## Discussion

This study investigated whether the SEMG amplitude and time frequency representations would be sufficiently sensitive to detect age specific differences in healthy individuals engaging in a 50% of maximum, fatiguing cyclic back extensions exercise. The findings of this research revealed that:

1. The load/weight administered in this task led to more perceived back muscle fatigue in the younger as compared to older individuals, but was only modest overall;
2. The RMS-SEMG onsets and fatigue related changes did not reveal relevant significant age or sex specific differences;
3. Despite comparable IMDF-SEMG onsets, the respective IMDF-SEMG fatigue related slopes were clearly less pronounced in the older as compared to younger participants, especially when the concentric portion of the cyclic exercise was considered, and
4. Between days’ re-test reliability of the SEMG variables revealed SEM and D-coefficient values that were comparable between old and young individuals with SEM values indicating low absolute reliability and D-coefficients indicating fair to excellent relative reliability.

### Individuals, maximum back extension strength, and mechanical performance parameters during the cyclic exercises

Overall, the older participants recruited in this study were more physically active than their younger counterparts. In fact, all older individuals clearly exceeded the recommended minimum physical activity levels [54]. Such high physical activity levels amongst the older individuals likely explains why the maximum back extension strength scores of our older participants were similar to those of our younger participants, and appeared to be 25 to 30% higher than would normally be expected [3]. Moreover, the scores obtained from our younger participants widely corresponded with those observed from healthy males and females who had been tested using similar equipment [55]. Therefore, the back scores obtained from the younger as well as the older participants who participated in this study likely reflect close to true maximum sores. The lack of any age specific differences in back extension strength further suggests that the consequences of an assumed aging-induced motor neuron loss in our older participants [8, 56] may have been functionally well compensated, most likely through their relatively high exercise levels.

The biomechanical variables “range of motion” and “mean movement velocity” that had been monitored to control the biomechanical quality of exercise performance were well matched with the overall requirements of the repetitive flexion extension test and kept constant throughout the exercise. Moreover, as neither the onsets nor the changes over time of these variables differed between the younger and older participants in a relevant way, no major confounders on EMG recordings from variable biomechanical performance of the cyclic exercise could be expected. Although the work performed during the exercise was comparable between the two age groups, the younger individuals reported significantly more back muscle fatigue at the end of the cyclic back exercise, even though the respective scores still indicated fatigue to be moderate. This result is inline with those from systematic reviews that found the back muscle endurance function to be better preserved in older as compared to younger individuals [4, 57]. In addition, findings from a study wherein healthy young individuals were able to perform a maximum of 34 cycles of back extensions on a Roman chair, with the back loaded to a similar extent and the cyclic exercise performed within the same range of movement and at comparable movement velocity [45], seems to externally validate the fatigue ratings observed amongst the younger participants in this study.

### RMS-EMG accompanying the cyclic back extension exercises

Research that investigated age specific differences in motor unit control using the needle electromyographic method observed a proportionally higher number of motor units activated that fired at similar [58] or modestly lower rates [8, 59] when older individuals produced a sub-maximum force comparable to that of younger persons. Against our expectations, this study’s results revealed a lack of relevant age-specific differences for the normalized back extensor RMS-SEMG onset score. Our results suggest that this variable is likely not sensitive enough to detect aging induced adaptive alterations in neuromuscular activation. Several confounders known to affect the RMS-SEMG provide an explanation. Among these, amplitude cancellation that preferentially occurs if motor unit action potentials (MUAPs) of simultaneously active motor neurons are more homogeneous in shape and have a longer duration [60, 61] may have significantly confounded age specific differences for this EMG variable. As with muscles of the extremities, aging back muscles are probably also composed of motor units whose MUAPs are, if compared to young muscles, larger in area and amplitude and more homogeneous in shape but reduced in absolute number [3, 6, 8, 9, 26, 56]. These characteristics expose the amplitude SEMG variables, resulting in a higher risk for amplitude cancellation with lower than expected normalized values in older than younger individuals.

As was found in previous studies [45, 62], the RMS-SEMG significantly increased over the course of the concentric phases of the exercise and tended to be more pronounced in the younger than in the older participants, when the concentric mode of the exercise was considered. This consistent result supports the theory that such fatigue related increases in SEMG amplitude reflect both a progressive recruitment and an increase in firing rate of motor units in order to compensate for fatigue induced muscle fiber contraction failure [63–65]. The back muscles of the younger participants in this study had a comparatively smaller percentage of motor units activated when moving the 50% of maximum load, followed by a relatively stronger pronounced progressive recruitment of motor units and a larger increase in firing rate. This was likely due to a higher reserve capacity to adjust for an increase in fatigue driven compensatory excitatory drive to the alpha motor-neuron pool in younger individuals. These factors could theoretically explain the more pronounced rise in SEMG amplitude in the younger participants. However, the differences in RMS-EMG increases failed to demonstrate sufficient age specific discriminative power. Therefore, this variable may, as with the amplitude SEMG onset variable, be assumed to be of minor importance if the early detection of aging muscle function is of interest.

It is interesting that, when the eccentric contraction mode of the exercise was considered, a significant decrease rather than an increase in RMS-SEMG was observed over the course of the exercise for both the elderly and younger group of participants. In eccentric contractions muscle fibers have a greater intrinsic force capacity [66] and the generated force is smaller than the load that is to be resisted. This is accompanied by recruitment of fewer MUs that fire at lower rates than during concentric contractions, if the muscle length vs. force relationship is comparable [67–69]. A more pronounced increase in amplitude cancellation with the duration of exercise in the older as compared to younger participants could likely be due to the fatigue-induced widening in the duration of the MUAPs, as well as a progressive recruitment of motor units that would be more homogeneous in shape in elderly persons with muscle fatigue.

### IMDF-EMG accompanying cyclical back extension exercises

In static back extensions at increasing strength, the median of the frequency spectrum (MF) only increased up to 30–50% of maximum [70]. If the strength further increases, then the MF-SEMG decreases. This is in a clear contrast to muscles of the upper and lower extremities whose mean MF-SEMG spectrum is known to increase with increasing muscle force production until progressive motor unit recruitment has finished [70, 71]. Considering all of the following: 1) a close relationship between median frequency of the EMG signal to the muscle fiber conduction velocity, 2) the shape of the action potential, and 3) the temperature of the muscle, such atypical changes of the MF-EMG from back extensors with increasing force would be best explained by the recruitment of the in-cross section larger slow twitch fibers at lower force thresholds than the smaller fast-fatiguing muscle fibers [35, 71, 72]. Likewise, confounding variables of the MF-EMG such as spatial filtering, differences in recruitment ranges of different muscles detected in the electrode recording area overlying muscles, and interference phenomena of the many concurrently active MUAP trains may further explain non-linear force- MF-SEMG behavior in increasing strength of back extensor muscles. As this study did not reveal any age or sex specific significant differences for the IMDF-EMG onset values, this variable likely provides a useful measure for normalizing the IMDF-EMG fatigue slopes. Such a normalization procedure would control for: 1) overall smaller muscle fiber conduction velocities of the older muscles; 2) other confounders of the SEMG signal including variability in subcutaneous fatty tissue under the electrode [64]; 3) age specific differences in maximum recruitment thresholds as well as differences in maximum recruitment thresholds between the short, deep, and longer superficial back extensor muscles [73]; and, 4) the loss of orderly recruitment of motor units, with larger motor units who fire at lower rates being recruited earlier in a contraction when force increases [6, 9].

The only SEMG variable that clearly indicated aging-induced muscle functional changes in cyclically activated back extensor muscles was the IMDF-SEMG fatigue slope. This result corresponds with our previous observation made from static back extensions that had been performed at 80% of maximum for 30s [24]. It is important to note that such age specific differences in SEMG spectral compression occurred although the load of the cyclic exercise equated only to that of 50% of maximum, and the older patients’ back muscle strength had been comparable in the two different age groups. If the fatigue dependent degree of compression of the IMDF-SEMG spectrum toward lower frequencies was mainly dependent on the slowing in muscle fiber conduction velocity, then an overall smaller percentage of fast fatiguing motor units had contributed to the significantly less pronounced IMDF-SEMG slopes in our older individuals. The flatter IMDF fatigue slopes observed from older adults were likely due to either: 1) adaptive changes in back muscles resulting from an increasingly forward-leaning posture of the trunk in people older than 50 years old [74] with an increased mechanical loading of these muscles during moving around, and/or 2) compensatory adaptive processes caused by an aging induced loss of motor neurons. For the latter explanation, orphaned fast twitch fibers that remain from the apoptotic neurons of previous high threshold motor units will be re-innervated by the axons of low threshold motor units, which are typically comprised of fatigue resistant muscle fibers. Such re-innervation would force a formerly fast fatiguing, yet re-innervated muscle fiber to adapt its behavior through altered mechanic demands towards those required for low threshold motor units. If such re-innervated fast twitch, fast fatiguing muscle fibers could not translate towards a slow twitch, fatigue resistant one [75], then apoptosis caused by overuse might occur. Indeed, co-expression of two instead of one myosin heavy-chain (MHC) isoforms has been demonstrated in at least 40% of the total muscle fibers within a muscle, [76] which suggests that such a fast to slow muscle fiber translation is likely an adaptive process. However, such a compensatory re-innervation after axonal degeneration may remain widely incomplete [36], as shown from the vastus lateralis muscles where 40% of muscle fibers had been lost in persons aged approximately 75 years old. The maintenance of the maximum back extension torque despite loss of fast twitch muscle fibers in our older participants may be explained through regeneration via the muscle fiber satellite cell pool, thereby further enlarging the motor units and compensating for lost muscle fibers. This would be consistent with research that found failure to expand the motor units to be associated with a loss in muscle mass [27], as well as research that muscle fiber atrophy can be overcome at least in part by physical exercise [77–79] and drug interventions [80]. It is worthwhile noting that with advancing age, regeneration through the type II fiber satellite cell pool seems more limited than that of type I muscle fibers [81]. In addition, more mechanic stress would train the enlarged motor units of older individuals who would comprise less glycolytic type II muscle fibers (but more aerobic type I and hybrid type II a muscle fibers), and thus increase the intrinsic twitch force output the muscle fibers of the remaining motor units [82].

The IMDF–SEMG fatigue slopes appeared to be more sensitive to detecting age specific differences during the concentric than in the eccentric phases of the exercise. The SEMG accompanying concentric versus eccentric contraction has been extensively discussed in a recent study [47]. In short, less recruitment of motor units and a lower firing rate during the eccentric phase of the contraction (as indicated by a reduced RMS SEMG) seems to lower the age specific discriminative power of the IMDF-SEMG slopes. This result suggests that a minimum threshold load relative to maximum may exist for conducting a diagnostically accurate test. In addition, our findings further suggest that normalization of the IMDF-EMG slope to the initial IMDF frequency is advisable, as it would compensate for confounders that may occur from spatial filtering due primarily to individual differences in the distance from the muscles to the surface electrodes.

### Reliability of SEMG variables

This study also investigated the reproducibility of the variables of interest and found the relative day-to-day retest reliability for the SEMG variables to be good to excellent, depending on the recording site. Absolute reliability, however, was highly variable for both the normalized slopes and the EMG onset values. This was true for both age specific subgroups. These observations concur with those from earlier research where healthy individuals performed cyclic free lifting [34] or a sustained 80% of maximum contractions [24]. Future research will have to address the need to improve the precision of the IMDF EMG fatigue variables or introduce new IMDF fatigue-based variables if the SEMG cyclic back extension exercise test is intended to be further developed to a sensitive standard biomarker to detect the very early cases of sarcopenia.

### Limitations

One may suspect that the fatigue slopes observed from our older population were biased by significantly higher physical activity scores as assessed with the IPAQ and higher than expected back extension strength scores. It is worth noting that the spectral MF-SEMG fatigue method is independent from an individual’s absolute maximum strength score, but the submaximum load needs to be derived from a true maximum performance. Given this, the age specific differences observed with this method would be widely unaffected by the training state of the back muscle unless the respective strength and power scores were considered in addition. This is important because training induced compensation in strength and power decline following an aging related loss of glycolytic type II fibers is mainly achieved through mechanisms leading to an increase in mechanic output in less fatigable type II and fatigue resistant type I muscle fibers [5, 77]. This all likely suggests that a decline in the proportion of high threshold motor units with age as indicated by less IMDF-SEMG fatigue would be widely unaffected by the training state of an individual’s back extension strength. This conclusion seems supported by very recent findings from age specific differences in SEMG-fatigue observed in cLBP patients a static 80% of maximum sustained isometric back extension during 30s. In this recent study, older cLBP patients were able to produce 85% MVC back extension strength of that observed from younger patients, however, older and younger cLBP patients were comparable in their physical activity scores as assessed with the IPAQ [83].

It could be argued that differences in blood perfusion of the back extensor muscles confounded the findings from the spectral SEMG observed in this study [64]. We did not monitor blood perfusion, nor did we take any muscle probes that would have confirmed a similar vascularization between age groups. However, this confounder seemed well controlled by the relatively highly trained state of the back muscles observed amongst our older healthy participants. The high level of training amongst older participants caused a capillarization of muscle fibers that was as good as that observed in young individuals [84]. However, even if back muscles had been clearly weaker, less trained and thus probably less capillarized in our older participants, the IMDF-SEMG slopes - as opposed to the IMDF onsets - would have remained widely unaffected as evidenced from individuals with peripheral occlusive disease [85, 86]. Thus, the age specific discriminative power of this variable has only a small risk of being confounded by altered capillarization of the back extensors.

It is also possible that sex specific differences could interfere with the IMDF-SEMG fatigue changes. In fact, fast fatiguing high threshold motor units of the back muscles were found to be smaller in females than males [87], and for both the RMS and IMDF fatigue SEMG recorded during static sustained back extensions, sex specific differences have repeatedly been observed in static sustained back extensions [88]. In this study, however, we did identify sex specific differences within slopes of the IMDF-EMG of the most negative electrode only; this suggests that the age specific effects observed for the IMDF-EMG in this study are widely unaffected by sex differences.

We attempted to record a full set of EMG data during the cyclic exercise from a total of 87 individuals. Unfortunately, artifact free recording from all electrode sites was feasible in only 56 individuals. Intermittent loss of electrode skin contact leading to major EMG artifacts, which occurred most frequently at the L5 and L2 recording sites, was one of the main reasons for exclusion of data. This may have reduced the statistical power to detect age specific differences in this explorative study. We therefore analyzed the fitted EMG data that considered side related differences in pairwise electrode recordings. Results between primary and secondary analyses were similar. In fact, future research will have to compare different types of electrodes with defined inter-electrode distance that would be unlikely detach during the performance of such a cyclic exercise.

### Implications

Loss of neurons may start in an individual’s later 40s, and seems to be independent from any muscle training performed [3]. As back muscle loss has been demonstrated to occur at a comparatively high rate [19], and impaired lumbar extensor muscle function (weakness and fatigue) is associated with loss of balance, postural alignment and control [20, 21, 23], and osteoporosis [89], functional assessment based on strength and endurance scores is of utmost importance. Proving the early loss of back-extensor muscles with a simple test like ours, before a decrease in back muscle strength and endurance becomes overt, seems key to early interventions intended to decrease the higher risk of future falls and frailty. However, before this functional back muscle test can be offered as a preventive diagnostic tool, data processing would need to be further developed to a fully automatic procedure, and the absolute reliability of the IMDF-EMG fatigue slope-based variables needs to be further improved. As our novel EMG-based test is based on a sub-maximum cyclic exercise protocol at moderate load, testing can likely be considered safe even in patients at risk for increased vertebral fractures, and it is simple and quick to perform [90]. Therefore, this test would meet the basic requirements for implementation of nation-wide screening to detect early forms of deterioration in muscles that are the most likely to predispose elderly persons to future falls and fall-related injuries, frailty and early death.

## Conclusions

Among the different SEMG variables recorded from a cyclic back extension exercise using a load equal to 50% of maximum and performed with a slow movement velocity, the IMDF-SEMG slope demonstrated clear age-specific effects in a reliable way. This result was observable despite the fact that back extension strength was comparable between the two age groups. The RMS-EMG variable appeared less discriminative. Thus, the IMDF-EMG fatigue method possesses great potential for further development toward a feasible biomarker intended to detect early signs of sarcopenic back muscle function.

## Data Availability

Anonymized data are made available upon request.

## Abbreviations

A/D: Analog/digital
ANOVA: Analysis of Variance
BMI: Body Mass Index
D: Coefficient of dependability
EMG: Electromyography
G-Theory: Generalizability Theory
ICC: Intraclass Correlation
IMDF: Instantaneous Median Frequency
IPAQ: International Physical Activity Questionnaire
MF: Median Frequency
MUAP: Motor unit action potential
MVC: Maximum Voluntary Contraction
RMS: Root Mean Square
ROM: Range of Motion
SEM: Standard error of measurement
SEMG: Surface Electromyography
VAS: Visual Analog Scale
